# Unusual case of Hashimoto’s encephalopathy and pseudo-obstruction in a patient with undiagnosed hypothyroidism: a case report

**DOI:** 10.1186/1752-1947-8-296

**Published:** 2014-09-06

**Authors:** Irfan A Shera, Anurag Vyas, Mohd Shafi Bhat, Qayser Yousuf

**Affiliations:** 1 Department of General Medicine Rama Medical College Hospital & Research Centre, Ghaziabad 245304, UP, India; 2 Department of Orthopaedics Rama Medical College Hospital & Research Centre, Ghaziabad 245304, UP, India; 3 Department of Advanced centre for Human Genetics, Sher-i-Kashmir Institute of Medical Sciences (SKIMS), Srinagar 190011, Kashmir, India

**Keywords:** Hashimoto’s encephalopathy (HE), Overt hypothyroidism, Pseudo-obstruction

## Abstract

**Introduction:**

Hashimoto’s encephalopathy is a relatively rare condition associated with an elevated concentration of circulating serum anti-thyroid antibodies, and is usually responsive to steroid therapy. However, hypothyroidism is a rare cause of pseudo-obstruction so here we present a case report of Hashimoto’s encephalopathy with gut pseudo-obstruction in an undiagnosed hypothyroid patient.

**Case presentation:**

A diagnosis of unknown aetiology of encephalopathy with gut dysmotility in an undiagnosed profound hypothyroidism case associated with cognitive decline and behavioural disorder was made in a 60-year-old Indian man. The associated clinical and laboratory features led to the final diagnosis of overt hypothyroidism with Hashimoto’s encephalopathy with gut pseudo-obstruction.

**Conclusions:**

Hashimoto’s encephalopathy is a rare disorder presenting with acute or sub acute encephalopathy of unknown aetiology so there are considerable chances of misdiagnosing it. The unusualness of this case is that since hypothyroidism is a rare cause of intestinal pseudo-obstruction, and presented concomitant with Hashimoto’s encephalopathy, that itself is a rare entity. Intestinal pseudo-obstruction is a potentially serious complication that must be recognized and treated promptly with adequate thyroid hormone therapy.

## Introduction

Hypothyroidism is a disorder caused by hypofunction of the thyroid gland. Iodine deficiency is the most common cause of hypothyroidism worldwide; however, in areas of iodine sufficiency Hashimoto’s thyroiditis and iatrogenic causes are most common. Because of its autoimmune nature there is a gradual decline in thyroid function with presentation of a wide range of disease symptoms. Some patients may have minor symptoms, which is called subclinical hypothyroidism, whereas others have a fall in unbound T4 levels and a steep rise in thyroid-stimulating hormone (TSH)>10μIU/L, which is referred to as clinical or overt hypothyroidism [1].

Lord Brain in 1966 described Hashimoto’s encephalopathy (HE) in a patient with Hashimoto’s thyroiditis as characterized by cloudiness of consciousness, tremors, cognitive loss and stroke-like episodes [2]. Since then HE has gained importance in differential diagnosis of encephalopathy of unknown origin. Shaw [3] in 1991 coined the term HE by describing the constellation of symptoms such as seizure, disorientation, frequent episodes of alternating hemiparesis, high protein levels in cerebrospinal fluid (CSF) and electrocardiogram (ECG) abnormalities. However, these patients also had hypothyroidism and positive anti-thyroid antibodies. Because of the severe neurological complexities the term HE is widely used while some other terms such as myxoedema madness [4], encephalopathy associated with autoimmune thyroid disease [5] or steroid responsive encephalopathy associated with autoimmune thyroiditis [6] have been discarded.

HE is a relatively rare condition; therefore there are considerable chances of misdiagnosing it. HE is generally considered to be an autoimmune encephalopathy; however the pathogenesis is still not clear. Antithyroid peroxidase (anti-TPO) antibodies are found in almost all cases of HE [7] but can also be present in the general population with normal thyroid function [8]. Moreover, it has been evaluated that there exists no direct causal relationship between anti-TPO antibodies and HE [9]. Hypothyroidism has frequently been associated with various gastrointestinal manifestations including constipation, bloating, flatulence, atrophic gastritis, ileus, atony and dilatation of oesophagus, stomach, gallbladder, small intestines and colon. Characteristic intestinal hypomotility in severe hypothyroidism may progress to intestinal pseudo-obstruction, paralytic ileus and megacolon [10]. Hypothyroidism is a rare cause of intestinal obstruction that can be reversed with thyroid hormone therapy.

Here we present a case report of HE with gut pseudo-obstruction. To the best of our knowledge this concomitant entity has not been reported to date.

## Case presentation

**Figure 1:**
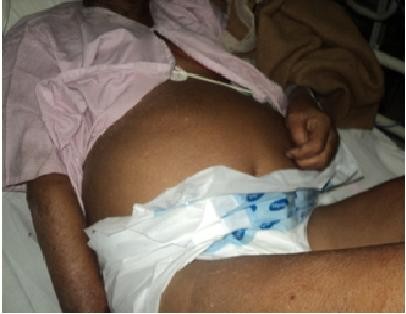
Figure showing distended abdomen.

**Figure 2:**
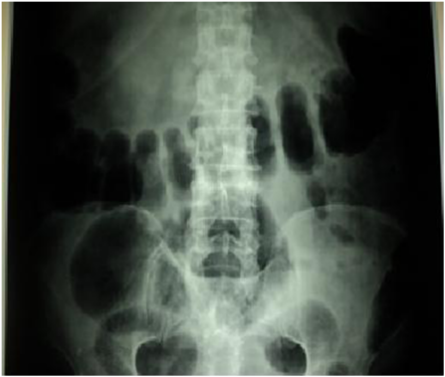
Supine abdominal plain film showing air-filled distended gut loops.

**Figure 3:**
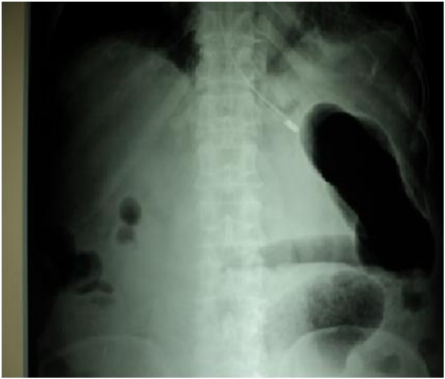
Upright abdominal plain film showing air-fluid level in dilated gut loops.

**Figure 4:**
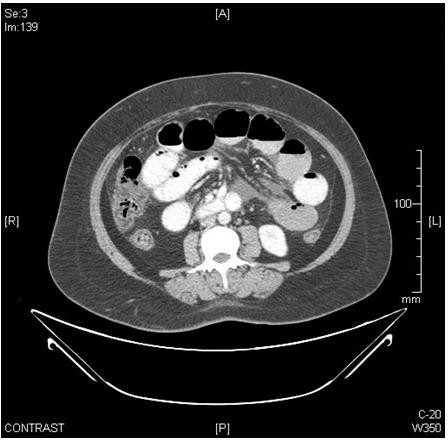
Axial contrast-enhanced computed tomography image shows dilated bowel loops.

**Figure 5:**
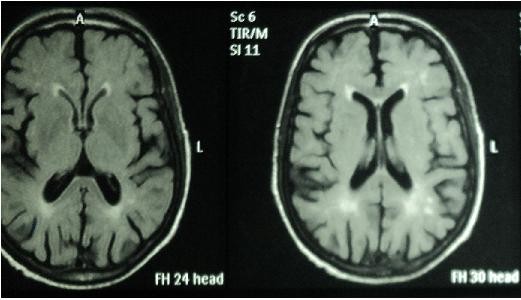
Fluid-attenuated inversion recovery sequence showing prominent bilateral cortical sulci and Sylvian fissures with bilateral periventricular ooze.

**Figure 6:**
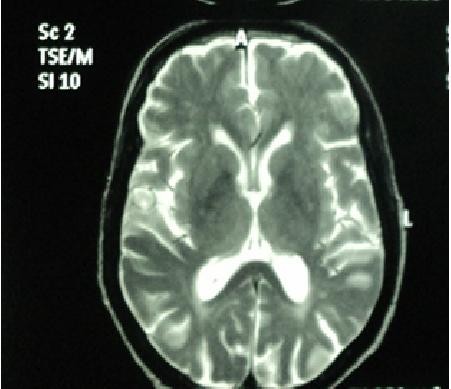
T2 magnetic resonance imaging image showing hyperintensities in periventricular areas.

A 60-year-old non-alcoholic, non-diabetic, normotensive Indian man of the state of Uttar Pradesh, working in printing press was brought to our emergency department with history of altered sensorium and abdomen distension of two days’ duration. There was history of slow mentation, cognitive decline characterized by inattention, and difficulty in finding words which prevented him from performing routine activities for the last three months. He also had behavioural disorder in the form of agitation, hallucinations and delusions of persecution. He had constipated bowel habits. There was no history of any drug intake.On examination he was drowsy. He had a hoarse voice, dry skin, puffy face, madarosis and cold extremities. He had a distended abdomen (Figure 1) with absent bowel sounds. He had sluggish deep tendon reflexes all over. Chest and cardiovascular examinations were normal.Laboratory investigations revealed macrocytic (mean corpuscular volume: 100) hypochromic anaemia (haemoglobin: 9.1g/dL) with leucopenia (total leukocyte count: 3900 per mm). His blood sugar, liver function test, kidney function test, arterial blood gas and electrolytes were within normal limits. His urine examination was normal. He had a sterile septic profile. A chest X-ray and ECG were normal. Abdominal ultrasonography revealed gaseous distention with dilated bowel loops. An X-ray of his abdomen showed features of obstruction with dilated gut loops and air-fluid levels (Figures 2 and 3). Axial contrast-enhanced computed tomography image of his abdomen showed dilated bowel loops (Figure 4). A whole body computed tomography (CT) scan was inconclusive. Erythrocyte sedimentation rate, C-reactive protein, antinuclear antibodies and human immunodeficiency virus serology were normal. Physical examination of his thyroid gland was unremarkable. A CT scan showed his thyroid gland to be diffusely atrophic with decreased attenuation. A thyroid profile revealed TSH of 76.52μIU/mL (normal: 0.39 to 3.55μIU/mL) with free thyroxine (FT4) of 0.1ng/dL (normal: 0.75 to 1.54ng/dL) and free triiodothyronine (FT3) of 0.42pg/mL (normal: 2.0 to 4.9pg/mL). His serum anti-TPO antibody level was 581 units/mL (normal: <60 units/mL). Magnetic resonance imaging (MRI) of his brain showed mild diffuse cerebral atrophy; fluid-attenuated inversion recovery (FLAIR) sequence showing prominent bilateral cortical sulci and Sylvian fissures with bilateral periventricular ooze (Figure 5), T2/FLAIR hyperintensities in bilateral periventricular and sub-cortical white matter suggestive of chronic ischemic changes were found (Figure 6). An electroencephalogram was normal. CSF examination showed reactivity for anti-TPO antibodies with elevated proteins and normal sugar level.

Special investigations were carried out, such as glucose hydrogen breath test was normal, serum cobalamin level was 532ng/L (normal 180 to 1000ng/L), blood lead level was 2.19μg/dL (normal: <25μg/dL) and gut manometry showed change in the frequency of the slow wave oscillations of smooth muscle electric potential features suggestive of intestinal dysmotility.

Final diagnosis of overt hypothyroidism with HE with pseudo-obstruction was made. He received hydrocortisone 200mg/day IV for a period of seven days which resulted in a significant improvement in his clinical condition after the third day of treatment. He became alert, oriented with time, place and person, and responded well to verbal commands. His mini-mental score was 14/30; it was not possible to compute this score earlier because he presented with altered sensorium. The treatment was maintained by tapering the dose of prednisone over a period of six weeks. He received levothyroxine 100μg/day orally from the second day of his diagnosis. His pseudo-obstruction was managed conservatively with Ryles tube suctioning, proctoclysis enema, metoclopramide and erythromycin. His bowel sounds resumed and he passed flatus on the second day of his admission. However, his abdominal distension regressed slowly over a period of one month. One month after discharge, he had remarkable improvement in all parameters including memory and cognition that made it possible for him to return to routine work. His CSF was re-examined after six weeks of steroid therapy and it was normal. His mini-mental score improved to 28/30 at six weeks of his follow up. Currently he is on levothyroxine 75μg/day with latest thyroid profile of: TSH of 4.3μIU/mL, FT4 of 1.2ng/dL, FT3 of 3.45pg/mL and anti-TPO antibodies level of 85 units/mL.

## Discussion

Clinical features of hypothyroidism usually have an insidious onset and affect a wide range of organ systems before one can make perfect diagnosis of overt hypothyroidism. In our case our patient was not aware of symptoms until he presented with neuropsychiatric syndrome and features of gut dysmotility.

HE is a rare neuropsychiatric syndrome, more common in women, associated with serologic evidence of antithyroid antibodies,when other causes of encephalopathy are excluded [11]. Our patient presented with encephalopathy with gut obstruction that could have indicated infectious, inflammatory, neoplastic, toxic and metabolic aetiologies. The authors ruled out all possibility of close differential diagnoses such as chronic lead toxicity as he had occupational exposure to lead, megaloblastic anaemia, gut neoplasia with overwhelming sepsis and brain metastasis. The MRI of the brain of our patient showed mild diffuse cerebral atrophy, prominent bilateral cortical sulci and Sylvian fissures with bilateral periventricular ooze associated with white matter ischemic lesions. The diverse MRI features of HE can vary from normal appearance, ischemic lesions, demyelination and vasogenic oedema to atrophy [12]. Our patient’s elevated anti-TPO antibodies in his serum and CSF and his response to steroid therapy seemed appropriate to establish a certain diagnosis.

The responsiveness of steroid therapy was the cornerstone for diagnosing HE. It is obvious that he responded to hydrocortisone, although levothyroxine was started from day two. This can be justified from the fact that levothyroxine has a half-life of approximately seven days; it will take at least six to eight weeks for a patient to become euthyroid, before one could recognize effectiveness of therapy. Similarly his neuropsychiatric manifestation responded to steroids dramatically rather than to thyroid therapy. However, his abdominal distension regressed slowly over a period of one month, once he attained euthyroid state, which made us conclusive that pseudo-obstruction of gut was consequence of profound hypothyroidism that responded to thyroid replacement.

Hypothyroid patients often complain of constipation and their gastric emptying time may be significantly delayed [13]. Myxedema ileus uncommonly complicates the hypothyroid state and is a rare cause of intestinal pseudo-obstruction that can return to normal after the thyroid disorder is corrected [14]. Our patient had constipation and small intestinal transit was significantly slowed as revealed by gut manometry. Profound hypothyroidism can cause intestinal dysmotility, results in paralytic ileus and intestinal pseudo-obstruction. These features respond to the thyroid hormone replacement therapy gradually over time.

It was further observed that he had a hoarse voice that reflected fluid accumulation in his vocal cords and tongue. Moreover, typical features of hypothyroidism such as dry coarse skin, puffy face with oedematous eyelids and non-pitting pretibial oedema were present in our patient.

## Conclusions

HE is a rare disorder with varying clinical manifestations that should be considered in any patient presenting with acute or subacute encephalopathy of unknown aetiology. Hypothyroidism is a rare cause of intestinal pseudo-obstruction which is a potentially serious complication that must be recognized and treated promptly.

## Consent

Written informed consent was obtained from the patient for publication of this case report and any accompanying images. A copy of the written consent is available for review by the Editor-in-Chief of this journal.

## Abbreviations

anti-TPO: Antithyroid peroxidase; CSF: Cerebrospinal fluid; CT: Computed tomography; ECG: Electrocardiogram; FLAIR: Fluid-attenuated inversion recovery; FT3: Free triiodothyronine; FT_4_: Free thyroxine; HE: Hashimoto’s encephalopathy; MRI: Magnetic resonance imaging; TSH: Thyroid-stimulating hormone.

## Competing interests

The authors declare that they have no competing interests.

## Authors’ contributions

IAS managed the case report, drafted the manuscript and put forward the analysis and interpretation of data. AV participated in the design of the study and acquisition of data. MSB conceived of the study, and participated in its design and coordination and helped to draft the manuscript. QY helped to draft the manuscript and critical revision of the manuscript. All authors read and approved the final manuscript.
